# Suppressive myeloid cells are expanded by biliary tract cancer-derived cytokines in vitro and associate with aggressive disease

**DOI:** 10.1038/s41416-020-1018-0

**Published:** 2020-08-04

**Authors:** Michael B. Ware, Mohammad Y. Zaidi, Jennifer Yang, Michael K. Turgeon, Alyssa Krasinskas, Thomas A. Mace, Kaitlin Keenan, Matthew R. Farren, Amanda N. Ruggieri, Yiman Li, Chao Zhang, Zhengjia Chen, Gregory S. Young, Omar Elnaggar, Zheng Che, Shishir K. Maithel, Tanios Bekaii-Saab, Bassel El-Rayes, Gregory B. Lesinski

**Affiliations:** 1 Department of Hematology and Medical Oncology, Winship Cancer Institute of Emory University, Atlanta, GA USA; 2 Department of Surgery, Winship Cancer Institute of Emory University, Atlanta, GA USA; 3 Divisions of Medical Oncology and Gastroenterology, Department of Internal Medicine, The Ohio State University, Columbus, OH USA; 4 Department of Pathology, Winship Cancer Institute of Emory University, Atlanta, GA USA; 5 Biostatistics and Bioinformatics, Winship Cancer Institute of Emory University, Atlanta, GA USA; 6 Center for Biostatistics, The Ohio State University, Columbus, OH USA; 7 Mayo Clinic Cancer Center, Mayo Clinic, Phoenix, AZ USA

**Keywords:** Cancer microenvironment, Immunology

## Abstract

**Background:**

BTC is an aggressive disease exacerbated by inflammation and immune suppression. Expansion of immunosuppressive cells occurs in biliary tract cancer (BTC), yet the role of BTC-derived cytokines in this process is unclear.

**Methods:**

Activated signalling pathways and cytokine production were evaluated in a panel of human BTC cell lines. Human peripheral blood mononuclear cells (PBMCs) were cultured with BTC supernatants, with and without cytokine neutralising antibodies, and analysed by flow cytometry or immunoblot. A human BTC tissue microarray (TMA, *n* = 69) was stained for IL-6, GM-CSF, and CD33^+^S100a9^+^ cells and correlated with clinical outcomes.

**Results:**

Immunomodulatory factors (IL-6, GM-CSF, MCP-1) were present in BTC supernatants. BTC supernatants expanded CD33^dim^CD11b^+^HLA-DR^low/−^ myeloid-derived suppressor cells (MDSCs) from human PBMCs. Neutralisation of IL-6 and GM-CSF in BTC supernatants inhibited activation of STAT3/5, respectively, in PBMCs, with heterogeneous effects on MDSC expansion in vitro. Staining of a BTC TMA revealed a positive correlation between IL-6 and GM-CSF, with each cytokine and more CD33^+^S100a9^+^ cells. Increased CD33^+^S100a9^+^ staining positively correlated with higher tumour grade, differentiation and the presence of satellite lesions.

**Conclusion:**

BTC-derived factors promote suppressive myeloid cell expansion, and higher numbers of CD33^+^S100a9^+^ cells in resectable BTC tumours correlates with more aggressive disease.

## Background

Biliary tract cancers (BTCs) comprise a rare, heterogeneous group of malignancies with an average 5-year survival rate ranging from 8 to 10% for intrahepatic and extrahepatic bile duct cancer, respectively.^1^ In the United States, >7000 new cases of BTC are diagnosed annually.^2^ Recent evidence suggests the anatomic origin of BTC, specifically intrahepatic cholangiocarcinoma (ICC), extrahepatic cholangiocarcinoma (ECC), and hilar cholangiocarcinoma, categorises tumours into distinct subtypes.^3–5^ Despite advances in targeting genomic aberrations such as *IDH* and *FGFR* fusions in BTC,^6–8^ there has been limited investigation into targetable immune signatures within BTC.^3^ We suspect that BTC tumours may have distinct immune features that can be leveraged for nuanced treatment. In particular, we hypothesised that the inflammatory nature of this disease may promote suppressive myeloid cell expansion that acts to limit lymphocyte responses to BTC.

Although immunotherapy has limited impact on BTC,^9^ a report by Tran et al. demonstrated that adoptive transfer of tumour-infiltrating lymphocytes can mediate BTC regression.^10,11^ In a separate study investigating patients with high programmed cell death ligand 1 (PD-L1) expression in tumours, programmed cell death protein 1/PD-L1 blockade had efficacy as monotherapy.^12^ These data suggest that it is possible to elicit potent antitumour immunity against BTC. Indeed, some BTC patients can mount T cell immune responses against their tumours, although mechanisms mediating escape from immune recognition have been reported.^13^ A deeper understanding of immune–tumour interactions in BTC is necessary to develop novel therapeutic strategies against this aggressive malignancy.

Inflammation of the bile duct, autoimmune disorders, parasitic infections and exposure to alcohol or toxins contributes to BTC pathogenesis.^9,14^ These inflammatory conditions upregulate cytokines such as interleukin-6 (IL-6), granulocyte macrophage colony-stimulating factor (GM-CSF), and transforming growth factor-β (TGF-β), yet specific mechanisms by which these cytokines influence tumour development and progression in BTC have yet to be described. A number of reports suggest that IL-6 may act in an autocrine or paracrine manner to enhance BTC growth and survival.^15,16^ In models beyond BTC, IL-6 acts with other tumour or stromal factors to expand immunosuppressive cells. In particular, GM-CSF enhances expansion of myeloid-derived suppressor cells (MDSCs),^17–19^ while TGF-β can expand T regulatory cells (Tregs)^20,21^ and promote T helper type 17 (Th17) differentiation, both of which can mediate immune suppression.^22,23^ MDSCs are of particular interest, given their capability to limit T and natural killer cell function through production of reactive oxygen or nitrogen intermediates and depletion of key amino acids.^24–26^ In addition, investigations of MDSCs have revealed significant impacts of these populations on disease progression and metastasis, whereby MDSCs actually precede neoplastic cells to sites of metastasis and provide a hospitable environment for cancer growth.^27–32^ Characterisation of these soluble factors and cellular interactions may reveal viable targets for future immunotherapy strategies.

The Janus kinase/signal transducer and activator of transcription (Jak/STAT) pathway is an important mediator in the inflammatory response.^15^ STAT proteins are transcription factors that promote expression of distinct genes that differentially regulate cell growth, survival, and inflammation. STAT1 is typically associated with growth arrest and apoptosis. In contrast, STAT3 and STAT5 are associated with proliferation, resistance to apoptosis, and avoidance of antitumour immune responses. Constitutive STAT3 or STAT5 activation occurs in many tumours and is implicated in malignant progression.^33^ A limited number of studies confirmed that nuclear localisation of STAT3 was detectable in BTC patient tumours^34^ and associated with shorter survival.^35^ In myeloid compartments, STAT3/5 signalling regulates a phenotypic switch to promote immunologic sequelae, including expansion of MDSCs, M2 macrophages, and a shift in the balance of Treg/Th17 cells.^36–38^ We postulate cytokine-mediated STAT3/5 activation in BTC may lead to expansion of immune-suppressive cell populations and disease progression.

In the present study, we hypothesise that BTC-derived cytokines contribute to immunosuppression through distinct signalling pathways. We demonstrate human BTC cells produce a unique profile of soluble cytokines, capable of inducing in vitro expansion of functional MDSCs. IL-6 and GM-CSF excreted from BTC cells contribute but likely act in concert with other factors to facilitate these changes in myeloid cells. Within human BTC tissue samples, we demonstrate elevated IL-6 and GM-CSF are associated with higher infiltration of CD33^+^S100a9^+^ myeloid cells. In addition, increased percentages of CD33^+^S100a9^+^ cells in BTC tumour tissue correlated with higher tumour grade, the presence of satellite lesions, and more poorly differentiated tumours. Taken together, our studies indicate a dynamic tumour promoting interaction between BTC and MDSCs, by which tumour cells drive MDSC expansion and contribute to aggressive disease characteristics. These data provide novel insight into the role for myeloid cells in resectable BTC and a greater understanding of cytokine-regulated mechanisms contributing to the ability of BTC to escape immune recognition.

## Methods

### Cell culture

Human SNU-245 and SNU-478 cell lines were purchased from the Korean Cell Line Bank (Seoul, Korea) and authenticated prior to receipt. Early passage cells were cultured in RPMI-1640 media (Gibco, Gaithersburg, MD, USA) containing 10% foetal bovine serum (FBS), 10 mM L-glutamine, and Antibiotic-Antimycotic (Thermo Fisher Scientific). Human BTC cell lines HuCCT1, HuH28, and WITT were a gift from Dr. Tushar Patel (Mayo Clinic, Jacksonville, FL), and Mz-ChA-1 was a gift from Dr. Shannon Glaser (Texas A&M Health Sciences Center, Bryan, TX).^39^ These cells were authenticated through ATCC cell line authentication service (Kit #135-XV). HuCCT1 and HuH28 cells were cultured in RPMI-1640 media (Gibco) containing 10% FBS, 10 mM L-glutamine, and antibiotics. Mz-ChA-1 cells were cultured in CMRL1-media (Gibco) containing 10% FBS, 10 mM L-glutamine, and antibiotics.

### Analysis of cytokines and chemokines in BTC culture supernatants

A panel of 18 cytokines and chemokines was analysed in BTC culture supernatants (harvested from 6-well plates when 70–80% confluent) using commercially available, high-throughput Luminex Multiplex Cytokine Kits (Procarta Cytokine Assay Kit, Affymetrix, Santa Clara, CA, USA). All samples were batch run in duplicate and quantified based on a standard curve for each analyte.

### Cytokine level validation by enzyme-linked immunosorbent assay (ELISA)

Supernatants from BTC cultures were validated for monocyte chemoattractant protein-1 (MCP-1), IL-6, GM-CSF, and granulocyte colony-stimulating factor (G-CSF) using ELISA (R&D Systems, Inc., Minneapolis, MN, USA). Samples were run in duplicate per the manufacturer’s recommendations.

### Phenotypic analysis of human immune cells

Antibodies for myeloid or T cell surface staining were as follows: mouse anti-human CD11b-PE (Bear1, IM2581U) or mouse IgG1 PE (isotype control) (679.1Mc7,IM0670U), mouse anti-human CD33-APC (D3HL60.251, IM2471U) or mouse IgG1-APC (679.1Mc7, IM2475U), mouse anti-human CD15-FITC (80H5, IM1423U) or mouse IgM-FITC (GC323, IM1269U), HLA-DR-PE-Cy7 (Immu-357, A40579) or mouse IgG1-PE-Cy7 (679.1Mc7, 6607099), or mouse anti-human CD8-APC (B9.11, IM2469U) (Beckman Coulter, Brea, CA, USA). Cells were incubated on ice for 30 min, washed, and fixed in phosphate-buffered saline (PBS) containing 1% formalin for flow cytometric analysis on a FACS Calibur or LSRII (BD Biosciences, San Jose, CA, USA).

### T cell-suppression assay

T cell-suppression assays were conducted as described.^24^ Briefly, CD3^+^ T cells were enriched from source leukocytes by negative selection with Rosette Sep reagents (STEMCELL Technologies, Inc., Vancouver, BC, Canada). T cells were labelled with 1 µM carboxyfluorescein succinimidyl ester (CFSE; Invitrogen, Grand Island, NY, USA) and cultured with CD3/CD28 beads (Invitrogen) for 3 days. Cells were collected, stained for CD8^+^ T cell markers, and fixed for flow cytometric analysis. Events were gated on CD8^+^ T cells and percentage of proliferation was determined based on CFSE dilution.

### IL-6 and GM-CSF neutralisation assay

To block the effects of IL-6 and GM-CSF, cultured BTC supernatants, or media containing recombinant IL-6 (10 ng/mL) plus recombinant GM-CSF (10 ng/mL), were pre-incubated with anti-IL-6 antibody (Ab; 5 µg/mL, clone 6708, R&D Systems), anti-GM-CSF Ab (10 µg/mL, clone 3209, R&D Systems), or both for 30 min prior to addition to peripheral blood mononuclear cell (PBMC) cultures as described.^40^ Phosphorylated STAT3 and MDSC differentiation were assessed as described above.

### Western blot analysis

Immunoblots were prepared as described^41^ using Abs for STAT3 (79D7, #4904), pSTAT3 (Y705, #9145), STAT5 (#9363), pSTAT5 (Y694, 9351), pSTAT1 (Y701, #9171), β-actin (#4967) (Cell Signaling Technology, Danvers, MA, USA), or STAT1 (#610185) (BD Transduction Labs, San Jose, CA, USA). Following incubation with horseradish peroxidase-conjugated secondary Ab, immune complexes were detected via SuperSignal® West Pico Chemiluminescent Substrate (Thermo Scientific, Waltham, MA, USA).

### Clinical data acquisition and tissue microarray (TMA) construction

All studies on human tissue were conducted under Institutional Review Board-approved protocol at Winship Cancer Institute of Emory University. A retrospective chart review was performed from patient electronic medical records to obtain demographic, perioperative, histopathologic, and clinical outcome data for patients included within the TMA analysis (*n* = 69). Alcoholism was defined as alcohol use resulting in mental or physical disorder, and tobacco use was defined as any patient-reported tobacco consumption in the perioperative setting as obtained from the medical record. All patients underwent curative-intent resection of biliary tract malignancy between the years of 2000 and 2015. Patients received postoperative surveillance cross-sectional imaging per the NCCN guidelines. Specifically, abdominal and pelvic computed tomography (CT) or magnetic resonance imaging with chest CT was obtained every 6 months postoperatively for 2 years and then annually up to 5 years.^42^ Overall survival (OS) was defined as time from resection to death. Recurrence-free survival (RFS) was defined as time from resection to radiographic evidence of recurrent disease. Patients who died prior to disease recurrence were excluded. Median follow-up time was 27.6 months. The TMA was constructed using formalin-fixed, paraffin-embedded (FFPE) tissue slices from resected patient samples (*n* = 69); descriptive parameters and clinicopathological data for these patients can be found in Supplementary Tables 1 and 2. Several replicate slides containing slices of 1–3 samples from each patient were constructed.

### Immunofluorescent staining of FFPE tissues

TMAs were stained for IL-6, GM-CSF, and CD33^+^S100a9^+^ cells as a tissue-based surrogate of MDSC as published by Ortiz et al., with modifications.^43^ After rehydration and antigen recall in citrate buffer (Sigma Aldrich, St. Louis, MO, USA), slides were blocked with 5% BSA in PBS and incubated overnight at 4 °C with Abs to IL-6 (mouse 1:200, SantaCruz, Dallas, TX, USA), GM-CSF (1:100, Abcam), or S100a9 (rabbit 1:200, Abcam) and CD33 (mouse 1:100, SantaCruz) diluted in blocking buffer. Control slides were used to ensure that no background from primary or secondary Abs was present. After overnight primary incubation, slides were washed and incubated with secondary Abs to mouse (donkey anti-mouse 1:1000 Alexa 488, Abcam, Cambridge, UK) or rabbit (donkey anti-rabbit 1:1000 Alexa 568, Abcam) for 1 h at room temperature. Slides were stained with 4,6-diamidino-2-phenylindole (DAPI) at 1:5000 and washed, followed by 30-min incubation in 0.1% Sudan Black B in 70% ethanol to quench background fluorescence. Slides were washed with 0.02% Tween-20 in PBS, and coverslips were mounted using Vectashield Hardset (Burlingame, CA, USA). Images were taken using a Leica SP8 confocal microscope at ×20 (IL-6 and GM-CSF) or ×40 (CD33^+^S100a9^+^). *Z*-stacks were collected of CD33^+^S100a9^+^ cells, with 4 images collected over 3 µm.

### Analysis of immunofluorescent images

Immunofluorescent images of TMAs were analysed using FIJI (NIH) and CellProfiler.^44^ For IL-6 and GM-CSF images, a MaxEntropy threshold for DAPI, IL-6 (AlexaFluor 488) and GM-CSF (AlexaFluor568) was determined using FIJI. Total area of particles within the threshold was measured and area of IL-6 and GM-CSF was normalised to DAPI for each image. Utilisation of thresholding allowed for exclusion of background signal. Max projection images of CD33^+^S100a9^+^ cells were analysed using CellProfiler. Thresholding was performed for intensity and size. DAPI, S100a9, and CD33 were identified as primary objects and related using DAPI as the parent object, with S100a9 and CD33 as children. DAPI parent objects with both S100a9 and CD33 children were counted as dual positive cells and represent a phenotype consistent with MDSCs as described by Ortiz.^43^ Counts for CD33^+^S100a9^+^ cells were normalised to total cell counts per image as determined by DAPI object identification. Each sample was imaged as a single ×20 image for IL-6 and GM-CSF and two ×40 sets of *Z*-stacks for MDSCs.

### Statistical analysis

For in vitro studies, descriptive statistics for CD33dim cells generated by BTC supernatants were reported. The mean and standard deviation were calculated and presented. The generalised estimating equation models were performed to test whether there were any significant changes by condition and treatment of the outcome. Multiple comparisons and tests were performed among the four treatments in each cell line condition. The significance level was set at 0.05. All analyses were conducted in SAS v9.4 (SAS Institute, Cary, NC, USA). In analysing phenotypes from TMA staining, *t* test was performed to compare differences in CD33^+^S100a9^+^ cell infiltration and IL-6 or GM-CSF staining between patient cohorts by relevant clinicopathologic factors. Spearman’s correlation coefficient was used to measure correlation of IL-6, GM-CSF, or CD33^+^S100a9^+^ cell measurements with tumour grade and tested with Wald’s test. For survival analyses, IL-6 and GM-CSF expression and CD33^+^S100a9^+^ cell infiltration were dichotomised at the median into two groups (high vs. low) among each biopsy sample. Survival functions for OS and RFS were estimated by Kaplan–Meier curves. Log-rank test was used to test differences in OS or RFS between cohorts.

## Results

### Human BTC cells secrete immunomodulatory cytokines

**Fig. 1:**
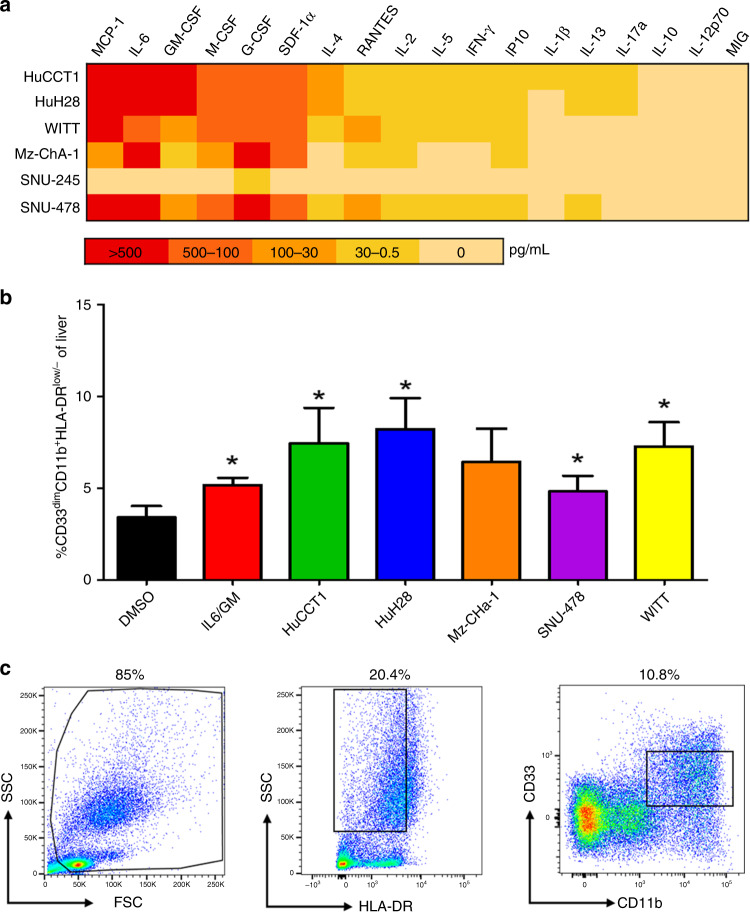
Secretion of soluble factors and in vitro differentiation of immunosuppressive MDSCs by BTC cell lines. **a** Heat map to summarise secretion of cytokines and chemokines from a panel of BTC cell lines. BTC cell lines were grown to 70–80% confluency, at which point supernatants were harvested and analysed using a multiplex platform. Data in the heat map represent the mean pg/mL values from at least *n* = 2 individual experiments. **b** PBMCs from healthy adult donors were incubated with 10 ng/mL of IL-6/GM-CSF (positive control) or with 10% of culture media supplemented with supernatants from individual human BTC cell lines for 7 days. These cells were then harvested, stained for MDSC phenotypic markers, and analysed via flow cytometry. Error bars represent standard deviation across donors. Asterisk (*) denotes statistical significance as compared to paired DMSO-treated PBMCs. For each condition, a minimum of *n* = 8 donors were analysed; Supplementary Table 3 indicates an exact *n* for each condition. **c** The gating schema for flow cytometric analysis of cells with an MDSC phenotype. Gates and voltage were set using appropriate fluorochrome-conjugated isotype control antibodies.

The profile of 18 cytokines and chemokines secreted from a collection of human BTC cell lines derived from intrahepatic (HuCCT1, HuH28), extrahepatic (WITT/Sk-Cha-1, SNU-245), gallbladder (Mz-ChA-1), or ampullary tumours (SNU-478) was characterised via multiplex analysis in culture supernatants (Fig. 1a). Cytokines involved in MDSC expansion and migration or T cell biology were our area of focus. Cell lines demonstrated variable levels of cytokine secretion. Overall, there was limited secretion (<10 pg/mL) of canonical Th1-type cytokines (interferon-γ, IL-2) from BTC cells but detectable concentrations of Th2-type cytokines (IL-4, IL-5) that were variable across cell lines. Most cell lines secreted abundant cytokines involved in expansion of immune-suppressive myeloid cells, including IL-6, GM-CSF, G-CSF, and macrophage colony-stimulating factor (M-CSF). In addition, differential expression of chemokines that regulate immune cell trafficking (stromal cell-derived factor-1 (SDF-1), RANTES (regulated upon activation, normal T cell expressed, and secreted), MCP-1) were also detectable in supernatants from cell lines. The presence of IL-6, GM-CSF, G-CSF, and MCP-1 in BTC supernatants was further validated with ELISA kits (Supplementary Fig. 1).

### BTC supernatants promote cytokine-driven differentiation of functionally suppressive MDSCs in vitro

Given the profile of cytokines in BTC supernatants, we postulated that soluble factors from human BTC may promote myeloid cell expansion. A well-characterised in vitro system was employed in which PBMCs from healthy donors were stimulated with IL-6/GM-CSF or 10% BTC supernatants for 7 days.^17,24^ The resulting cells were stained to evaluate a phenotype consistent with MDSCs (CD33^dim^CD11b^+^HLA-DR^low/−^) and analysed by flow cytometry (Fig. 1b, c). Supernatants from 5 human BTC cell lines led to significant expansion (mean = 5.2-fold ± 1.52, all *p*s < 0.02) of CD33^dim^CD11b^+^HLA-DR^low/−^ cells in vitro. Notably, the only BTC line that failed to significantly expand MDSCs was Mz-ChA-1, which originated from a gallbladder tumour. BTC supernatants did not compromise human PBMC viability after culture during this time period, confirming observations that indicated a phenotypic shift (not shown). The number of samples analysed for each condition can be found in Supplementary Table 3.

To validate cells generated via stimulation with BTC supernatants were functionally suppressive, CD33^+^ cells isolated from these cultures via magnetic separation were incubated with autologous, CFSE-labelled, activated CD3^+^ T cells for 3 days at a 1:1 and a 1:2 ratio of CD3^+^ T cells to CD33^+^ myeloid cells. A significant reduction in CD8^+^ T cell proliferation was observed upon co-culture with CD33^+^ cells generated via both IL-6/GM-CSF (positive control) and BTC supernatants at either 1:1 or 1:2 ratios (all *p*s < 0.026). These data were reproducible using autologous T cells and in vitro generated MDSC from four donors (Supplementary Fig. 2).

### IL-6 and GM-CSF are predominant cytokines that induce MDSC expansion and activation of Jak/STAT signalling by BTC supernatants

**Fig. 2:**
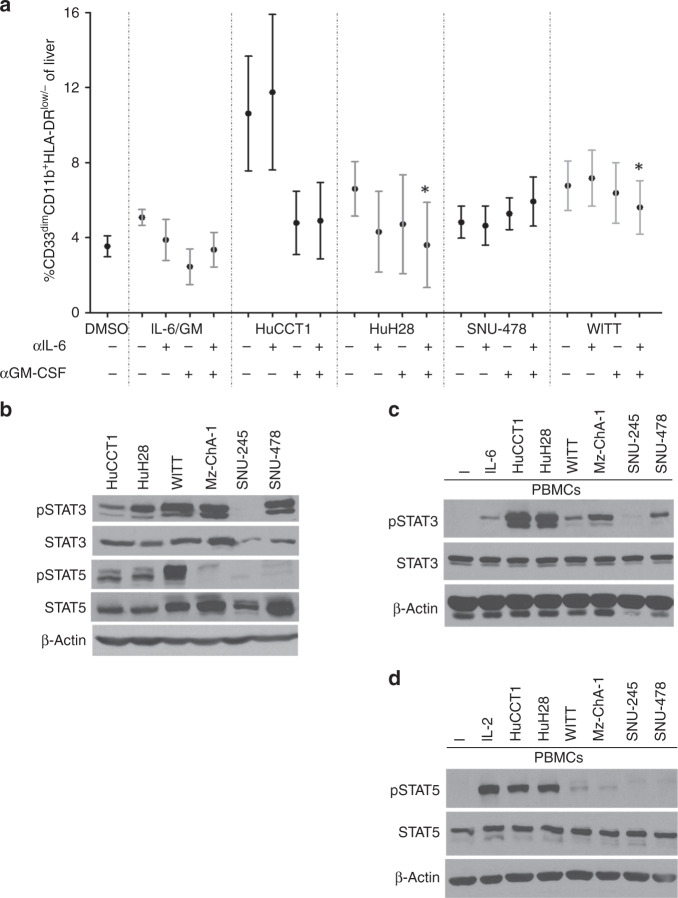
Cytokine neutralization in vitro alters MDSC expansion and inhibits Jak/STAT signaling in PBMCs induced by BTC supernatants. **a** PBMCs were stimulated with 10 ng/mL of IL-6/GM-CSF (positive control) or with 10% of culture media supplemented with supernatants from individual BTC cell lines ± IL-6 or GM-CSF neutralising antibody for 7 days. Cells were then harvested and stained for the MDSC phenotype and analysed via flow cytometry. Error bars represent standard error of measurement. Asterisk (*) denotes significance compared to GM-CSF neutralisation alone. For each condition, a minimum of *n* = 3 donors were analysed; Supplementary Table 3 indicates an exact *n* for each condition. **b** Basal activation of the Jak/STAT pathway in a panel of BTC cell lines. Immunoblot analysis was conducted to assess constitutive expression of phosphorylated STAT3 (Tyr^705^), STAT5 (Tyr^694^), and STAT1 (Tyr^701^). Levels of total STAT proteins and β-actin were included as loading controls. Data shown are representative from at least *n* = 3 individual experiments. **c** Healthy donor PBMCs were incubated for 20 min with culture media supplemented with 10% supernatants from individual BTC cell lines. Cells were then lysed and analysed by immunoblot. PBMCs showed increased pSTAT3 (Tyr^705^) and **d** pSTAT5 (Tyr^694^) following a 20-min incubation with BTC supernatants.

**Fig. 3:**
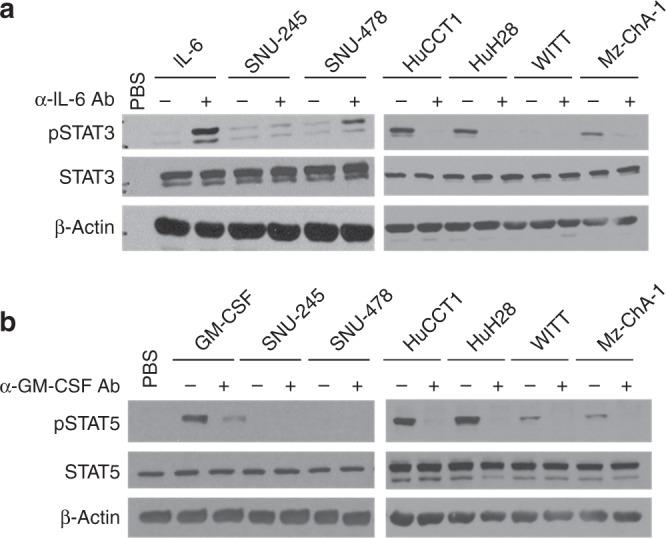
IL-6 and GM-CSF neutralisation of BTC supernatants inhibits activation of STAT3 and STAT5, respectively, in PBMCs treated with neutralised supernatants. Immunoblot analysis showing increased pSTAT3 and pSTAT5 in PBMCs after culture with BTC supernatants are abrogated by **a** neutralising Ab against IL-6 or **b** neutralising Ab to GM-CSF (α-IL-6 or α-GM-CSF). PBMCs stimulated with IL-6 and GM-CSF served as positive controls for pSTAT3 and pSTAT5, respectively. Data shown are representative of *n* = 3 individual experiments. β-Actin was used as a loading control. (−) = media alone, (+) = stimulation with IL-6 (10 ng/mL, positive control for pSTAT3) or GM-CSF (20 ng/ml, positive control for pSTAT5).

We next evaluated the BTC cell lines that made abundant IL-6 and GM-CSF to determine the roles of these cytokines in mediating MDSC expansion. Only a modest reduction in MDSC expansion occurred when GM-CSF was neutralised alone or in combination with IL-6 in supernatants from the HuCCT1 cell line (Fig. 2a) that was not statistically significant (*p* = 0.058 and *p* = 0.078, respectively). Similar data were obtained in other cell lines when GM-CSF alone or in combination with IL-6 were neutralised in supernatants from other cell lines; data trended towards an inhibition of MDSC expansion but with insignificant results (Fig. 2a). Of note, dual neutralisation of IL-6 and GM-CSF significantly decreased MDSC neutralisation compared to single-agent GM-CSF neutralisation in HuH28 and WITT cell lines (Fig. 2a; *p* = 0.001 and *p* = 0.019, respectively). This neutralising Ab approach had no effect on MDSC generation by supernatants from the SNU-478 cell line (Fig. 2a), which demonstrated moderate levels of many different cytokines (Supplementary Fig. 1A–F). The Mz-ChA-1 gallbladder line was omitted from this assay, as it was the only line that failed to significantly expand MDSCs (Fig. 1b). Consistent with the ability of IL-6 and GM-CSF to activate Jak/STAT signalling,^45^ phosphorylation of STAT3 and STAT5 was evident in five or three of the six BTC cell lines, respectively (Fig. 2b). In contrast, basal STAT1 phosphorylation was not detectable in any cell line (not shown). In separate experiments, BTC supernatants were applied to PBMCs and elicited signalling in trans (Fig. 2c, d). IL-6 was the dominant BTC-derived cytokine responsible for these signalling events, as Ab-mediated neutralisation of IL-6 abrogated most STAT3 phosphorylation (Fig. 3a) induced in PBMCs by BTC supernatants. In cell lines capable of inducing STAT5 phosphorylation in PBMCs, Ab-mediated neutralisation of GM-CSF was sufficient to disrupt this signalling event (Fig. 3b). This effective blockade of STAT3/5 activation in PBMCs further supports our data showing that IL-6 and GM-CSF from BTC lines contribute to expand MDSCs and indicate a distinct signalling axis involved in this phenomenon.

### Intratumoural levels of IL-6 and GM-CSF correlate with increased CD33^+^S100a9^+^ myeloid cell infiltration

**Fig. 4:**
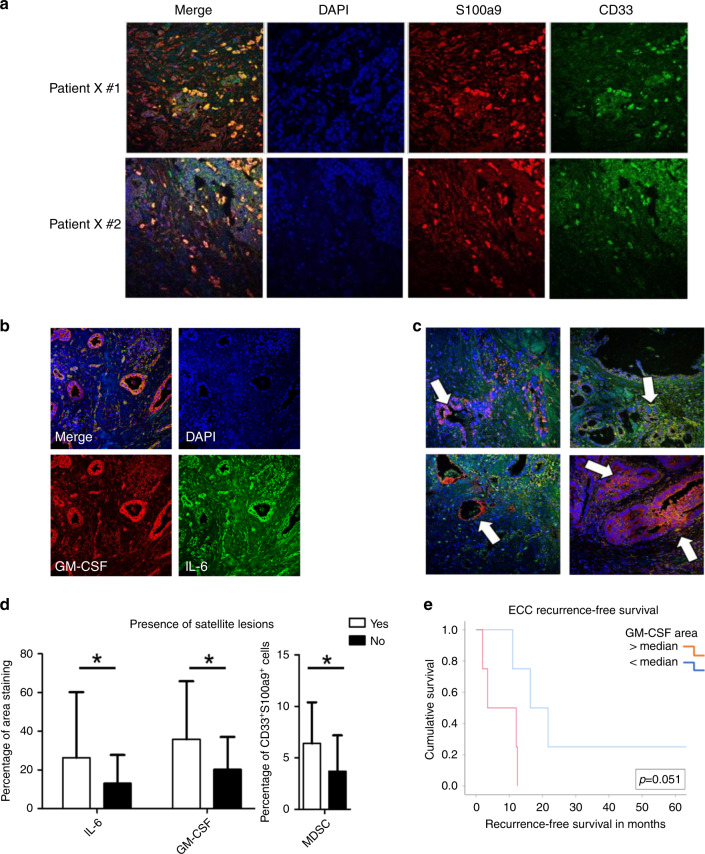
Immunofluorescent staining of biliary tract cancer tissue microarray and correlation of cytokine and cellular biomarkers with clinicopathological features of BTC. **a** Images (×40) of biliary tract cancer tissue microarray stained for DAPI (blue), S100A9 (red) and CD33 (green). Dual stained cells (yellow) are shown in merged image. **b** Images (×20) of patient tissue demonstrating high expression of IL-6 and GM-CSF in biliary tract cancer tissue microarray. Stained for DAPI (blue). GM-CSF (red) and IL-6 (green). **c** Representative images (×20) from 4 separate patients showing variability in IL-6 and GM-CSF staining, with white arrows indicating localisation of stains to ductal regions of tissue. **d** Graph demonstrating the correlation between increased staining for IL-6, GM-CSF and MDSC infiltration with the presence of satellite lesions. Significnace is indicated by an asterisk (*p* Value < 0.05) **e** Graph demonstrating the relationship between patients with GM-CSF staining above the median (red line) or below the median (blue line) and recurrence-free survival (*p* = 0.051).

A series of 69 BTC patient samples was obtained to define relationships between IL-6, GM-CSF, and myeloid biomarkers to clinical parameters (Supplementary Tables 1 and 2). Immunofluorescent staining (Fig. 4a–c) and analysis (Supplementary Fig. 3A–C) defined the distribution of CD33^+^S100a9^+^ cells, GM-CSF, and IL-6 between patients. No statistically significant differences for these markers were observed between ICC and ECC subtypes (*p* = 0.677 for GM-CSF; *p* = 0.119 for IL-6), although a trend was evident for MDSCs (*p* = 0.055). When correlating IL-6 with GM-CSF, there was a significant positive correlation between both cytokines in tumours which persisted in both ICC and ECC subtypes (Table 1, Supplementary Fig. 4). A significant correlation of IL-6 and GM-CSF levels with CD33^+^S100a9^+^ cell infiltration was also observed, which was more pronounced in the ECC subtype (Table 1). The CD33^+^S100a9^+^ cell population was phenotypically defined using Ab clones compatible for staining in FFPE tissue and identified in a prior study as consistent with an MDSC phenotype.^43^ Analysis of these biomarkers in the context of relevant clinicopathologic data revealed that higher CD33^+^S100a9^+^ cell infiltration, IL-6, and GM-CSF were each significantly associated with the presence of satellite lesions across patients (Fig. 4d and Table 2). Further, increased GM-CSF was associated with higher T stage, and CD33^+^S100a9^+^ cell infiltration was associated with higher tumour grade and less differentiated tumours (Table 2). When data were stratified by tumour subtype (ECC, ICC), there were no statistically significant correlations between IL-6, GM-CSF, or CD33^+^S100a9^+^ cell infiltration and clinicopathologic factors.

**Table 1:** Analysis of IL-6, GM-CSF, and CD33^+^S100a9^+^ staining of a human BTC tissue microarray revealed significant correlations of these soluble and cellular biomarkers with each other on both a subtype-specific and nonspecific level.

Correlation	Group	*R-*value (*p* value)
Percentage of MDSC and IL-6 area	All	0.18033 (0.0143)
	ICC	0.19178 (0.0329)
	ECC	0.37197 (0.0072)
Percentage of MDSC and GM-CSF area	All	0.28922 (<0.0001)
	ICC	0.24695 (0.0057)
	ECC	0.44586 (0.0010)
IL-6 area and GM-CSF area	All	0.76172 (<0.0001)
	ICC	0.71174 (<0.0001)
	ECC	0.87537 (<0.0001)

**Table 2:** Association of IL-6 expression, GM-CSF expression, and CD33^+^S100a9^+^ cell infiltration as measured by immunofluorescence with clinicopathologic features of patients within biliary malignancy tissue microarray (*n* = 69).

All patients	IL-6 expression		GM-CSF expression		CD33^+^S100a9^+^ infiltration	
Clinicopathologic variable	Mean area (±STD)	*p* Value	Mean area (±STD)	*p* Value	Mean area (±STD)	*p* Value
Disease recurrence						
Yes (32)	18.4 (25.7)	0.323	27.2 (23.3)	0.122	4.6 (3.9)	0.288
No (30)	13.1 (13.8)		18.9 (17.5)		3.5 (3.9)	
Lymph node positive						
Yes (12)	13.2 (12.1)	0.52	21.5 (20.1)	0.699	3.4 (3.4)	0.536
No (29)	17.9 (24.0)		24.2 (20.8)		4.3 (4.0)	
Tumour differentiation						
Well (3)	12.3 (13.0)	0.712	25.4 (15.4)	0.906	1.6 (1.5)	**0.050**
Moderate (40)	14.6 (16.3)		24.1 (20.0)		5.0 (4.0)	
Poor (20)	19.0 (28.6)		21.8 (22.5)		2.8 (3.0)	
Lymphovascular invasion						
Yes (22)	16.7 (28.9)	0.589	21.2 (25.9)	0.343	3.9 (4.0)	0.513
No (20)	20.8 (18.4)		27.8 (17.3)		4.7 (3.7)	
Perineural invasion						
Yes (18)	11.8 (9.6)	0.327	19.9 (16.5)	0.433	4.3 (3.7)	0.76
No (14)	16.8 (18.2)		25.1 (20.7)		4.7 (4.0)	
Tumour grade						
G1 (3)	12.3 (13.0)	0.659	25.4 (15.4)	0.375	1.6 (1.5)	**0.025**
G2 (36)	14.5 (17.0)		23.3 (20.1)		5.3 (4.1)	
G3 (24)	18.5 (26.3)		23.4 (22.0)		2.8 (2.8)	
T stage						
T1 (20)	13.8 (19.5)	0.065	24.5 (18.4)	**0.02**	4.5 (3.9)	0.916
T2a (8)	13.0 (10.3)		15.0 (5.6)		4.8 (3.5)	
T2b (12)	31.2 (34.6)		40.2 (30.4)		4.6 (4.3)	
T3/T4 (20)	12.3 (11.0)		18.7 (18.0)		3.8 (3.7)	
Satellite lesions						
Yes	26.4 (33.8)	**0.033**	35.9 (29.9)	**0.01**	6.4 (4.0)	**0.02**
No	13.1 (14.6)		20.3 (16.7)		3.7 (3.5)	

Significant values have been highlighted with bold text.

IL-6, GM-CSF, and CD33^+^S100a9^+^ cell infiltration were not associated with RFS or OS across patients (Supplementary Fig. 5A–F). However, stratification based on ICC or ECC revealed differences. While no relationship between these biomarkers and RFS or OS was evident in ICC patients (Supplementary Fig. 6A–F), there was a trend (*p* = 0.051) towards higher GM-CSF and worse RFS in ECC patients (Fig. 4e). In ECC patients, CD33^+^S100a9^+^ cell infiltration and IL-6 were not associated with RFS or OS, and GM-CSF was not associated with OS (Supplementary Fig. 7A–E).

## Discussion

Several redundant mechanisms limit immune recognition of tumours in patients with advanced BTC.^46^ This report explored the unique contribution of the cytokines IL-6 and GM-CSF and their relationship to signalling and myeloid cells in the context of BTC. We demonstrate that these BTC-derived factors expand functionally suppressive MDSCs in vitro and may have clinical implications when present in resectable patient tumours. These data are novel in the setting of BTC and aligned with their role in other gastrointestinal malignancies.^24,47^ Our results suggest that IL-6 and GM-CSF deserve investigation as therapeutic targets in BTC due to their ability to activate Jak/STAT signalling across tumour or immune cell types and their correlation with phenotypically defined myeloid cells in tumours from BTC patients.

This study lends credence to the concept that BTC has the capacity to exploit immune cells by secreting soluble factors that act in trans to drive signalling events, thereby influencing cell phenotype and function. Although IL-6 and GM-CSF emerged as the focus of this study, our data clearly point out that these factors are part of a larger imbalance encompassing multiple immune and metabolic factors in BTC. Certainly these factors can act in concert to facilitate MDSC expansion, along with other cell types including Tregs and Th17 cells.^24,48,49^ Further investigation of other BTC-derived factors may reveal cytokine/chemokine-induced expansion or migration of other immune subsets within the tumour microenvironment. Of particular interest for future studies are MCP-1, M-CSF, G-CSF, and SDF-1, all of which were secreted in abundance by BTC cells.

Considering our data, it is likely that IL-6, GM-CSF, or other cytokines in BTC are derived from multiple cell compartments. Indeed, fibroblasts, myeloid cells, and T cells can produce these cytokines and other immunomodulatory factors.^24,50,51^ In addition, the feedforward nature of the IL-6/Jak/STAT3 pathway can exacerbate cytokine production among any combination of these cellular subsets.^24,52–54^ This study also did not investigate Stromal Derived Factors known to be present in BTCs such as prostaglandin E_2_ and cyclooxygenase-2. This latter phospholipase has a key role in suppressing T cell-driven immune responses to cancer and driving MDSC expansion and promoting biliary cancer tumorigenesis.^55,56^ Given the dynamic relationship observed in the study between MDSCs and BTC, other targets such as these deserve further consideration. Here, we have specifically focussed on contributions of intratumoural IL-6 and GM-CSF, but further study is needed to assess tissue levels of other BTC-derived cytokines. Our data demonstrating activation of STAT3/5 in BTC cells and in PBMCs suggest a dual role for Jak/STAT signalling in promoting cancer cell proliferation and MDSC expansion. Further characterising the signalling dynamics in response to cytokines secreted by tumour cells may reveal novel targets for BTC that can be leveraged in combination with immunotherapy.

The analysis of patient tumours further established clinical relevance for IL-6, GM-CSF, and MDSC in BTC. Of note was the heterogeneous expression of these factors, and the fact that their expression was strongly correlated. The significant positive correlation between IL-6 and GM-CSF with infiltration of CD33^+^S100a9^+^ cells indicates that these cytokines contribute to progression by modulating the immune composition of the tumour. Of particular future interest is the significant correlation between the presence of MDSCs in pathologic specimens with aggressive tumour characteristics. The role of MDSC in establishing a pre-metastatic niche in various organs, particularly the liver, is an area of growing research with significant findings demonstrating mechanisms by which MDSCs shift local tissue microenvironments.^28,32,57,58^ The correlation observed here between worse tumour grade, poorly differentiated tumours, and the presence of satellite lesions demonstrates a relationship between aggressive tumour development and MDSCs. This correlation has also been observed in renal cell carcinoma, hepatocellular carcinoma, and other solid cancers.^27,31,59,60^ Given these data and the growing body of evidence suggesting that MDSCs play an integral role in patient responses to immune checkpoint blockade, further efforts to target the suppressive mechanisms of these cells in BTC should be made.^29,30,61,62^ Although these data are provocative, one limitation of this study is that the data are limited to a single phenotypic definition of CD33^+^S100a9^+^ cells.^43^ This is unfortunately due to lack of robust, validated immunohistochemical methods for MDSC phenotypic subsets in paraffin-embedded tissues. We are also aware that this population can encompass myeloid cells which may not functionally suppress antitumour immunity in BTC. It should also be noted that MDSC infiltration and cytokine staining in the tumours of these patients seem to independently correlate with clinicopathological features. Thus, while there is a direct correlation between increased staining for these cytokines and MDSC infiltration, the manner in which IL-6, GM-CSF, and MDSCs contribute to disease severity is likely complicated and influenced by other soluble mediators. One other factor to consider is the temporal aspect of this process that cannot be captured by a single time-point biopsies.

Stratification of patient samples into ICC and ECC subtypes revealed a trend towards a correlation between GM-CSF and worse RFS in ECC. While not significant, it is also notable that the average percentage of CD33^+^S100a9^+^ cell infiltration in ICC trended higher than in ECC. These data suggest that immune phenotypes may vary between ICC and ECC subsets of BTC. In contrast, the expression of IL-6, GM-CSF, or CD33^+^S100a9^+^ cells were not related to OS in these patients. These data are challenging to interpret but may reflect the limited study population of resectable patients, rather than metastatic BTC. The inherent characteristics of a resectable patient population may have limited the frequency of patients with pronounced differences in advanced disease, though these patients often undergo only diagnostic biopsy, leaving little residual tissue for research. Other limitations include retrospective design that poses a challenge when correlating TMA data with clinical outcomes, as some patients were lost to follow-up. We are aware of these issues but confident that this data set provides a valuable opportunity to examine a unique series of specimens in a relatively rare tumour type.

It is tempting to speculate that inflammatory changes leading to tumour progression are influenced by the anatomical location of BTC. To our knowledge, no human BTC cell lines are available from hilar cholangiocarcinoma, which would be an interesting comparator. Although this retrospective study is derived from one patient cohort, results suggest that further investigation into subtype-specific immune signatures may be revealing. While BTC can delineate into actionable subtypes for therapy (*FGFR2* fusion or *IDH* mutations), improved understanding of immune differences in BTC may aid design of immunotherapy trials.^63,64^ Previous research has pointed to tissue-specific influences on inflammation and immune response to disease and infection. Of note, recent evidence demonstrates that conjugated bile acids, found in liver bile, can influence T cell populations within the gut.^63,64^ Defects in pathways regulating sensing and efflux of bile by T cells can lead to imbalances of effector T cells and regulatory immune populations.^64^ We hypothesise that anatomical location may determine exposure of BTC to various bile acids and hepatic enzymes. These exposures may influence response of T cells, myeloid cells, and other immune populations to tumour-derived cytokines and growth factors, ultimately determining the composition of the tumour microenvironment.

In conclusion, this study characterised cytokine production of BTC cells and found IL-6 and GM-CSF as key cytokines that contribute to STAT signalling and MDSC expansion in vitro. We also demonstrated significant correlations with increased levels of these cytokines and CD33^+^S100a9^+^ cells in BTC tumours. Further, tumours with elevated numbers of CD33^+^S100a9^+^ cells were generally higher-grade tumours and poorly differentiated with detectable satellite lesions. These data indicate that tumour-derived IL-6 and GM-CSF may function as mediators of MDSC-driven immune suppression in BTC and strategies to modulate MDSCs deserve further investigation as therapeutic targets.

## Supplementary information

Compiled PDF of all Supplemental Data

## Data Availability

The data sets generated during and/or analysed during the current study is available from the corresponding author on reasonable request.
