# Supplementary material for: Suppressive myeloid cells are expanded by biliary tract cancer-derived cytokines in vitro and associate with aggressive disease
Source: Br J Cancer. 2020 Aug 4;123(9):1377–86. doi: 10.1038/s41416-020-1018-0 (PMC7591861; doi:10.1038/s41416-020-1018-0)
Supplement: Supplementary file 1 — Compiled PDF of all Supplemental Data [file 41416_2020_1018_MOESM1_ESM.pdf]

Supplemental Figure 1

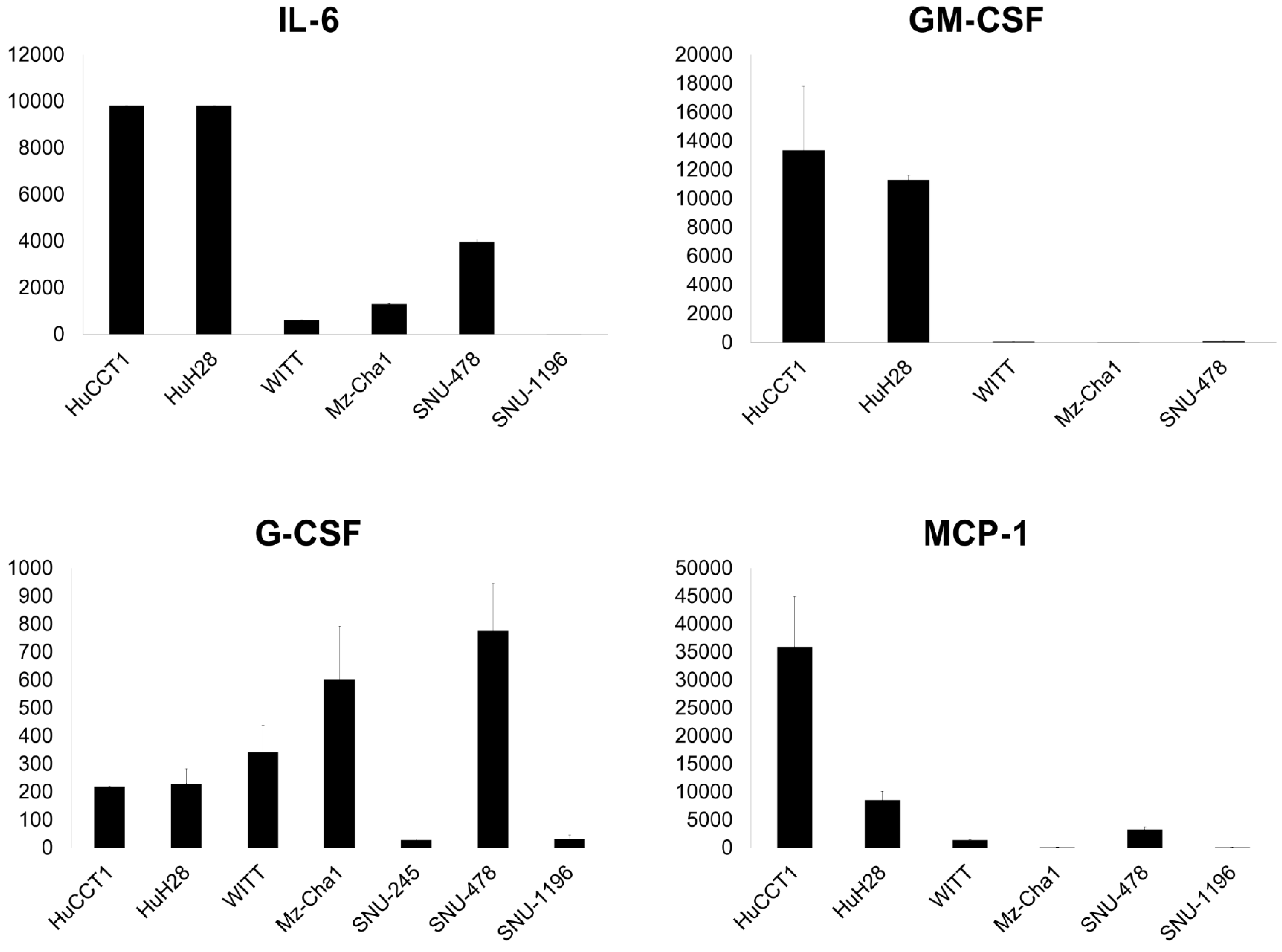

Supplemental Figure 1. ELISA quantification of cytokines, confirming Luminex results for the cytokines A) IL-6 B) GM-CSF C) G-CSF and D) MCP-1 in a panel of BTC cell lines. BTC cell lines were grown to 70-80% confluence, at which point supernatants were harvested and analyzed using ELISA kits. DATA shown represent at least n=3 biological replicates.

Supplemental Figure 2

A

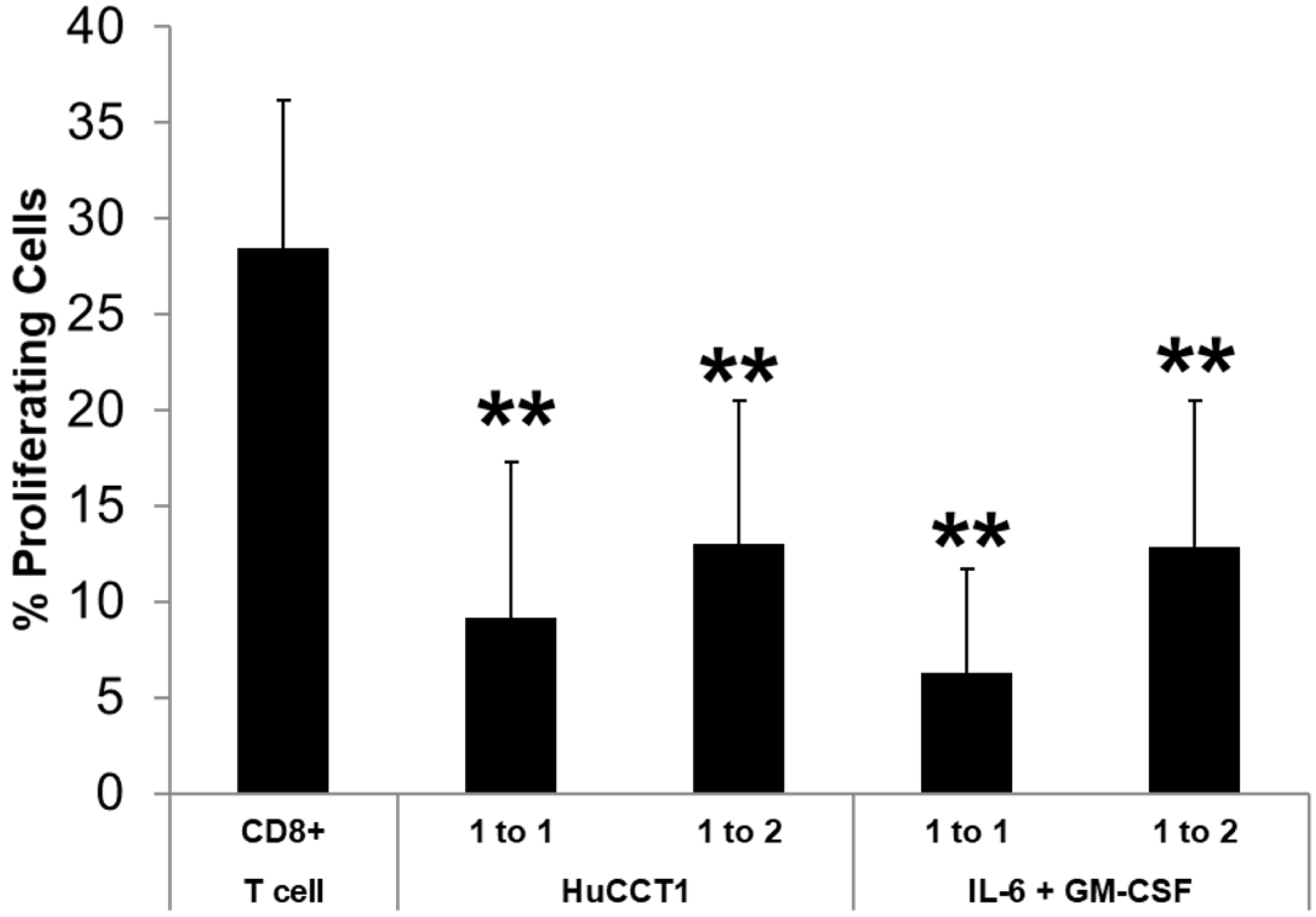

Supplemental Figure 2. A) Autologous T cells were labeled with CSFE and incubated with CD33-selected MDSCs for 3 days at a 1:1 or 1:2 ratios. HuCCT1 supernatant-generated MDSCs inhibited T cell proliferation significantly in $CD8^+$ T cells at 1:1 and 1:2 ratios ($p = 0.0069$ and $p = 0.026$, respectively). Culture of T cells with MDSC derived from culture of PBMCs with IL-6 + GM-CSF also significantly inhibited $CD8^+$ T cell proliferation at 1:1 and 1:2 ratios ($p = 0.0033$ and $p = 0.0223$, respectively). Error bars represent the standard deviation from experiments using PBMCs or T cells from n = 4 autologous donors with similar results. ** Denotes statistical significance as compared to $CD8^+$ T cells alone.

Supplemental Figure 3

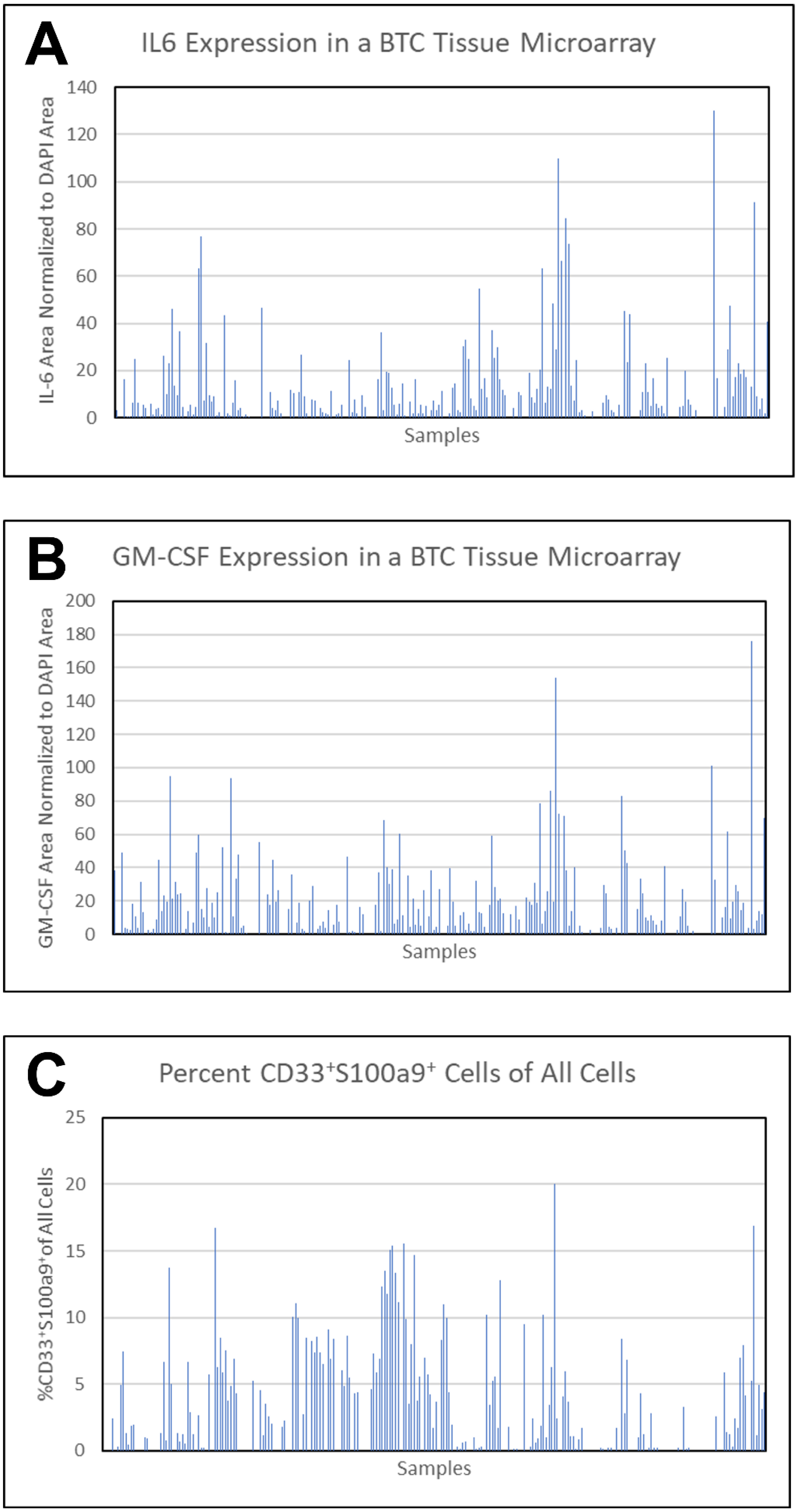

Supplemental Figure 3. A) Quantification of IL-6 staining in tissues from the microarray revealed high variability between patients. **B)** Quantification of GM-CSF staining in tissues from the microarray revealed high variability between patients. **C)** CellProfiler analysis of $CD33^{+}S100A9^{+}$ staining in patient tissues reveal high variability in the number of dual positive cells between patients, with most patients having detectable numbers of cells present in representative biopsies.

Supplemental Figure 4

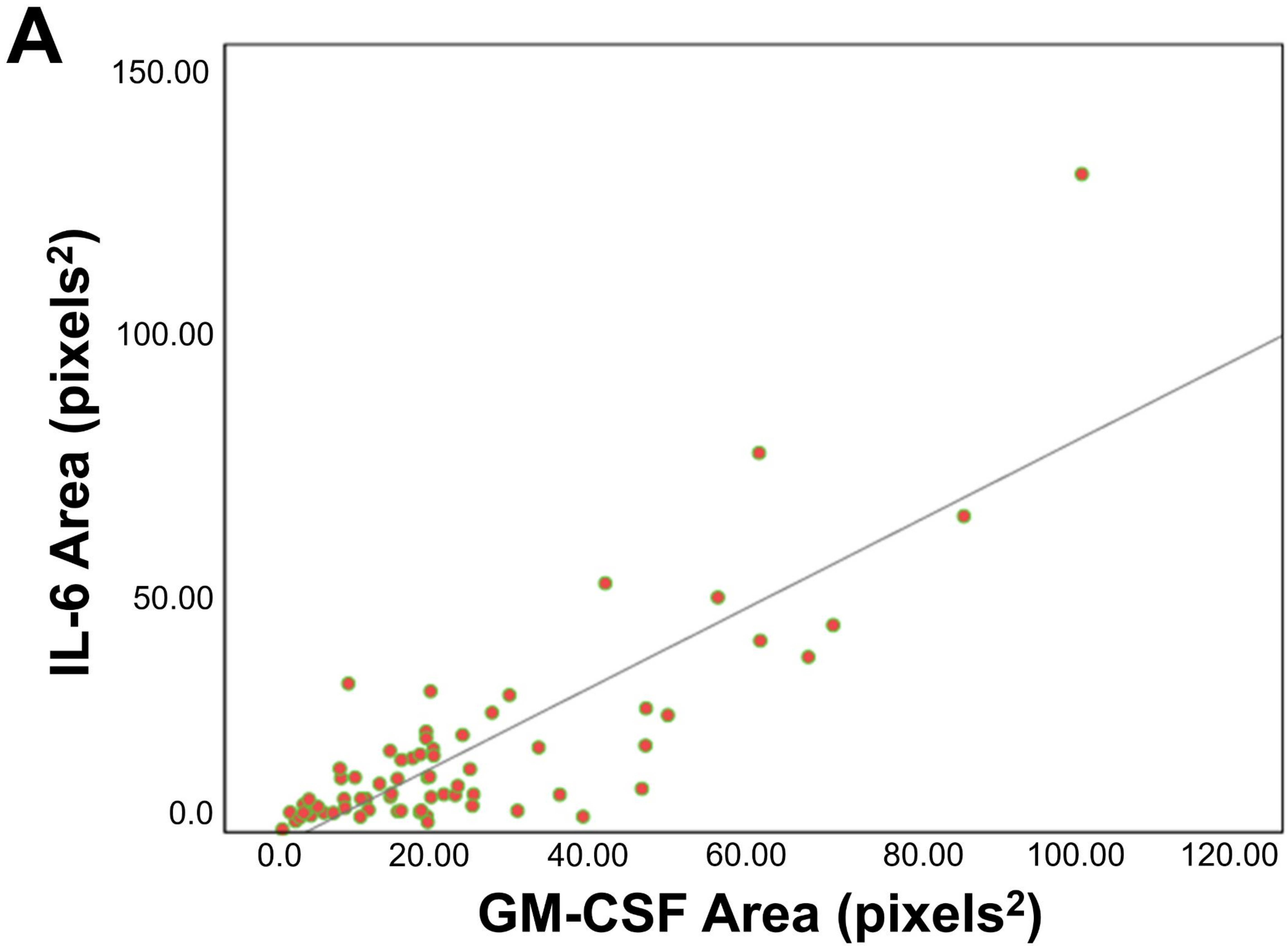

Supplemental Figure 4. A) Graph demonstrating the correlation of IL-6 and GM-CSF staining of samples from a BTC tissue microarray.

Supplemental Figure 5

All Patients

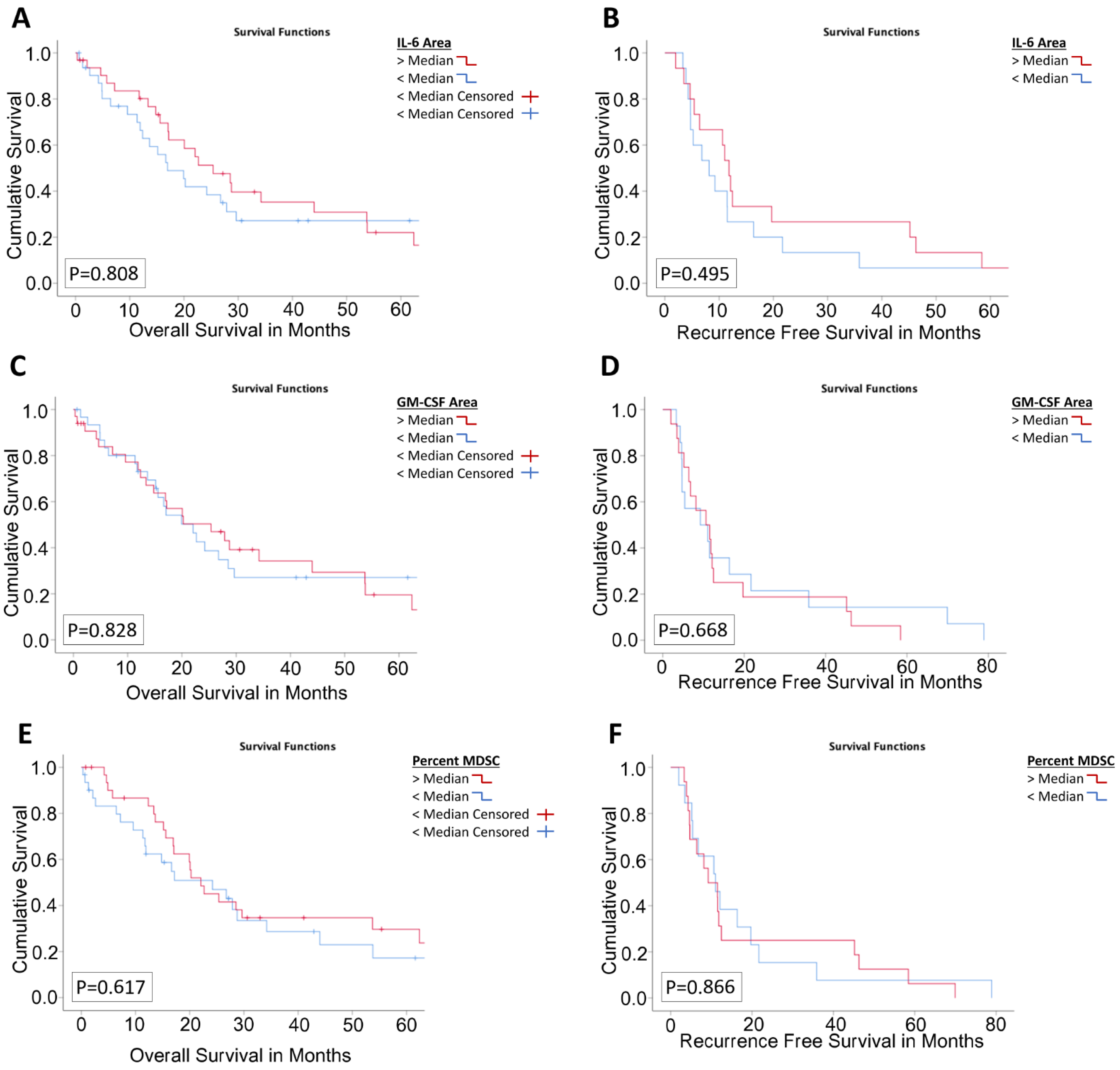

Supplemental Figure 5. Graph demonstrating the relationship between all patients with (A) IL-6 staining above the median (red line) or below the median (blue line) and overall survival or (B) recurrence free survival (C) GM-CSF staining above the median (red line) or below the median (blue line) and overall survival or (D) recurrence free survival (E) Percent MDSC staining above the median (red line) or below the median (blue line) and overall survival or (F) recurrence free survival (in months). P-values for each comparison are shown on individual graphs.

Supplemental Figure 6

ICC Patients

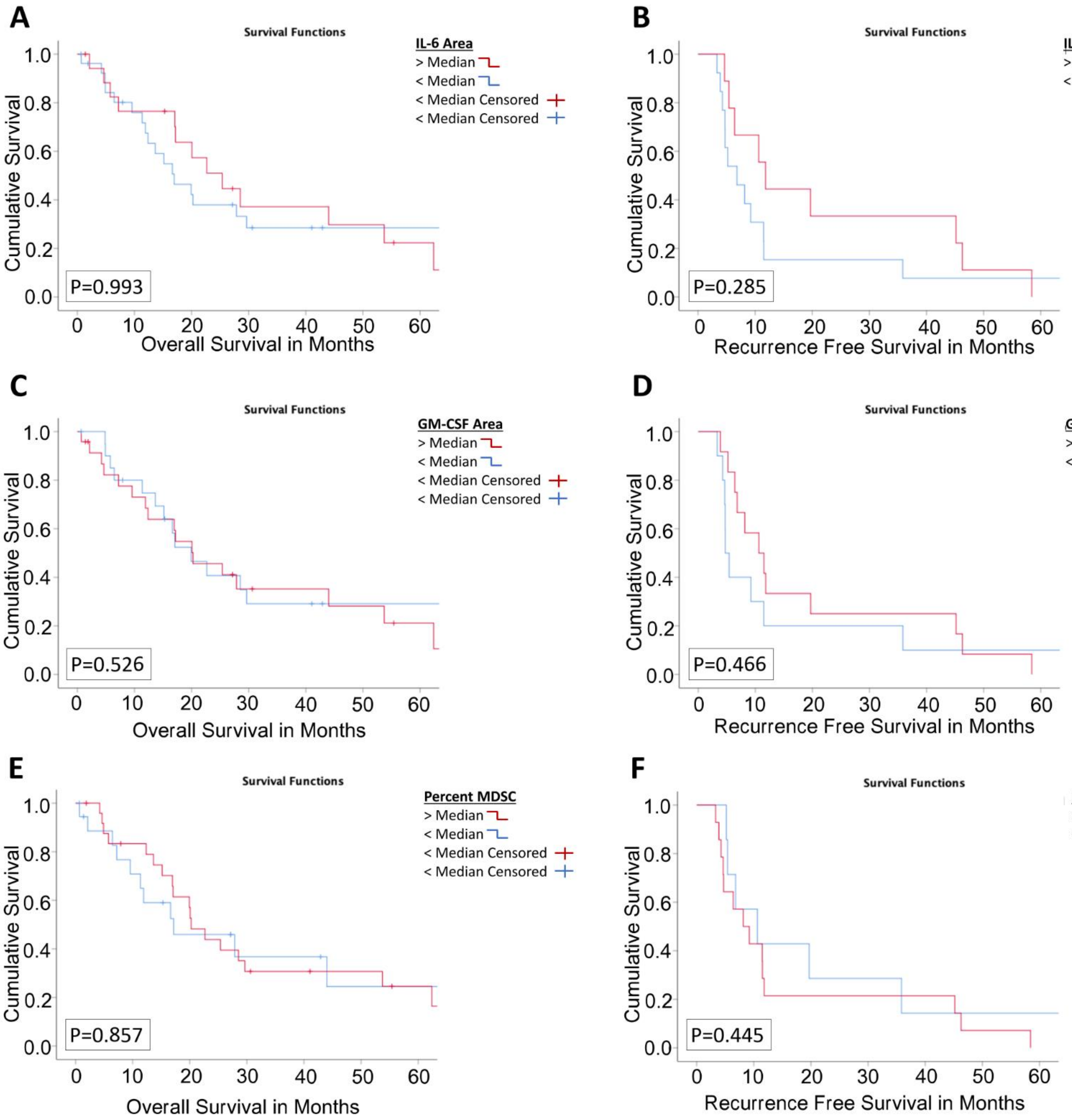

Supplemental Figure 6. Graph demonstrating the relationship between ICC patients with (A) IL-6 staining above the median (red line) or below the median (blue line) and overall survival or (B) recurrence free survival (C) GM-CSF staining above the median (red line) or below the median (blue line) and overall survival or (D) recurrence free survival (E) Percent MDSC staining above the median (red line) or below the median (blue line) and overall survival or (F) recurrence free survival (in months). P-values for each comparison are shown on individual graphs.

Supplemental Figure 7

ECC Patients

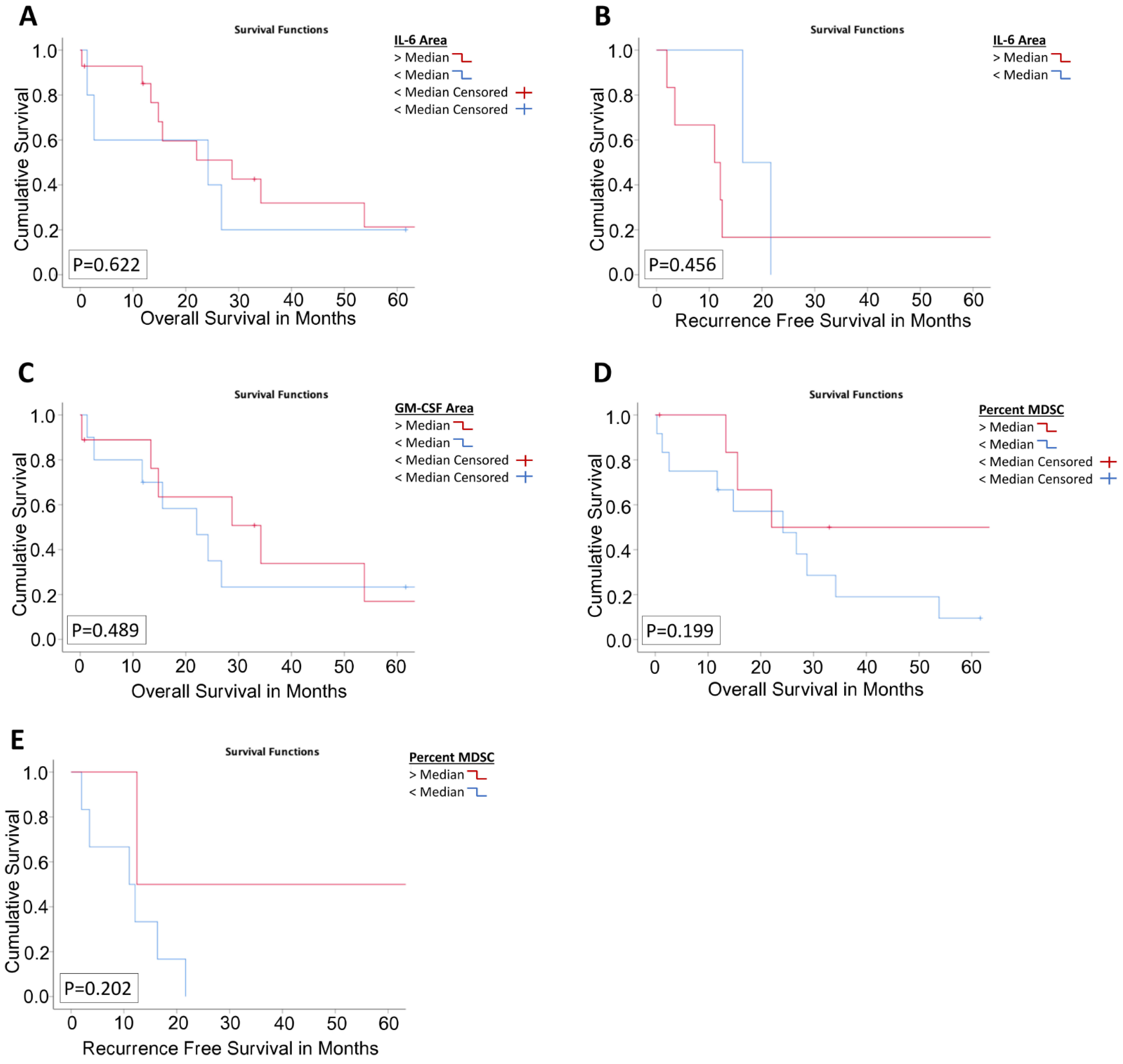

Supplemental Figure 7. Graph demonstrating the relationship between ECC patients with (A) IL-6 staining above the median (red line) or below the median (blue line) and overall survival or (B) recurrence free survival (C) GM-CSF staining above the median (red line) or below the median (blue line) and overall survival (D) Percent MDSC staining above the median (red line) or below the median (blue line) and overall survival or (E) recurrence free survival (in months). P-values for each comparison are shown on individual graphs.

Supplemental Table 1. Descriptive parameters of BTC patients represented on the BTC tissue microarray stained for IL-6, GM-CSF and CD33+S100a9.

Baseline Variable	n (%)
Age (years), mean, ± STD	62.4 ± 10.6
Male	27 (39.1)
BMI, mean, ± STD	30.3 ± 8.5
Race	
White	52 (75.4)
Black	8 (11.6)
Past Medical History	
Hepatitis B	6 (8.7)
Hepatitis C	8 (11.6)
Alcoholism	24 (34.8)
Tobacco Use	16 (23.2)
Location of Tumor	
Intrahepatic	45 (65.2)
Extrahepatic	19 (27.5)
Gallbladder	1 (1.4)

Supplemental Table 2. Pathological features of surgically resected specimens from patients whose tissue is represented on the human BTC tissue microarray.

Baseline Variable	n (%)
Tumor Size (cm), mean, ± STD	5.8 ± 3.8
Tumor Differentiation	
Poorly Differentiated	20 (29.0)
Moderately Differentiated	40 (58.0)
Well Differentiated	3 (4.3)
Tumor Grade	
G1	3 (4.3)
G2	36 (52.2)
G3	24 (34.8)
Lymphovascular Invasion	22 (31.9)
Perineural Invasion	18 (26.1)
Lymph Node Positive Disease	12 (17.4)
T Stage	
T0	1 (1.4)
T1	19 (27.5)
T2a/T2b	20 (29.0)
T3	18 (26.1)
T4	2 (2.9)
Median Overall Survival	20.1 months
Median Recurrence-free Survival	11.0 months
Mean Follow-up	27.6 months

Supplemental Table 3. Number of donors for each condition represented in Figure 1B and Figure 2A

Condition	Number of Donors Analyzed
DMSO	21
IL6/GM-CSF	28
IL6/GM-CSF anti-GM	7
IL6/GM-CSF anti-GM	4
IL6/GM-CSF anti-GM anti-IL6	4
HuCCT1	11
HuCCT1 anti-IL6	9
HuCCT1 anti-GM	3
HuCCT1 anti-GM anti-IL6	3
HuH28	8
HuH28 anti-IL6	5
HuH28 anti-GM	3
HuH28 anti-GM anti-IL6	3
SNU478	8
SNU478 anti-IL6	5
SNU478 anti-GM	5
SNU478 anti-IL6 anti-GM	5
WITT	14
WITT anti-IL6	8
WITT anti-GM	5
WITT anti-IL6 anti-GM	5
